# Orellanine specifically targets renal clear cell carcinoma

**DOI:** 10.18632/oncotarget.19555

**Published:** 2017-07-25

**Authors:** Lisa Buvall, Heidi Hedman, Alina Khramova, Deman Najar, Lovisa Bergwall, Kerstin Ebefors, Carina Sihlbom, Sven Lundstam, Anders Herrmann, Hanna Wallentin, Emelie Roos, Ulf A. Nilsson, Martin Johansson, Jan Törnell, Börje Haraldsson, Jenny Nyström

**Affiliations:** ^1^ Institute of Neuroscience and Physiology, Gothenburg, Sweden; ^2^ Institute of Medicine, Gothenburg, Sweden; ^3^ Institute of Clinical Sciences at University of Gothenburg, Gothenburg, Sweden; ^4^ National Food Agency, Uppsala, Sweden; ^5^ Department of Laboratory Medicine, Pathology, Lund University, Malmö, Sweden; ^6^ Proteomics Core Facility at Sahlgrenska Academy, University of Gothenburg, Gothenburg, Sweden

**Keywords:** clear cell renal cell carcinoma, nephrotoxin, anti-carcinogenic treatment, apoptosis, necrosis

## Abstract

Renal cell carcinoma (RCC), arising from the proximal tubule in the kidney, accounts for approximately 85% of kidney cancers and causes over 140,000 annual deaths worldwide. In the last decade, several new therapies have been identified for treatment of metastatic RCC. Although these therapies increase survival time compared to standard care, none of them has curative properties. The nephrotoxin orellanine specifically targets proximal tubular epithelial cells, leaving other organs unaffected. We therefore hypothesized that the selective toxicity of orellanine extends to clear cell RCC (ccRCC) cells since they emanate from proximal tubular cells. Orellanine would thus target both primary and metastatic ccRCC *in vitro* and *in vivo*. We found that orellanine induces dose-dependent cell death in proximal tubular cells and in all ccRCC cells tested, both primary and cell lines, with no toxicity detected in control cells. The toxic action of orellanine involve decreased protein synthesis, disrupted cell metabolism and induction of apoptosis. In nude rats carrying human ccRCC xenografts, brief orellanine treatment eliminated more than 90% of viable tumor mass compared to control rats.

This identifies orellanine as a potential treatment concept for ccRCC patients on dialysis, due to its unique selective toxicity towards ccRCC.

## INTRODUCTION

Renal cell carcinoma (RCC) is a widespread malignancy with over 350,000 cases reported annually, causing 140,000 deaths per year [1, 2]. One in five patients present with advanced disease already at diagnosis and 30 % of those initially diagnosed with localized disease will develop metastases, even after successful removal of the original tumor [2, 3]. For metastatic disease the 5-year survival is 20% and the median survival time is close to one year [3]. Numerous therapeutic options have been suggested [4, 5] but the outcome for patients with metastatic disease or with locally advanced tumors remains poor [2, 3]. Recently developed therapies include targeting of vascular endothelial and platelet growth factors, mammalian target of rapamycin and receptor tyrosine kinases. These therapies have shown moderately longer progression-free survival compared to standard of care [6–8]. While such a respite is undoubtedly of crucial importance to the individual patient, it is clear that modern medicine stands without curative treatment options against metastatic RCC. The majority of renal cancers (75%), referred to as clear cell RCC (ccRCC), evolve from the epithelial proximal tubular cells within the kidney with few cases indicating other tubular origin [2].

Orellanine (3,3΄4,4΄-tetrahydroxy-2,2΄bipyridine-1,1΄-dioxide), is a nephrotoxin produced by mushrooms of the *Cortinarius* family or ‘deadly webcap’ [9]. Renal histopathology after orellanine ingestion demonstrates progressive damage selectively to proximal tubular cells in rats and in humans, but no evident glomerular damage [10]. The mechanism of action of orellanine includes formation of ROS both *in vivo* and *in vitro* and reduction of antioxidant enzymes [11]. Renal failure is normally apparent on the third day after ingestion with no other symptoms or signs of lesions in other organs. Thus, the single clinical outcome after accidental orellanine intoxication is irreversible renal failure, with patients becoming dependent on dialysis treatment [9, 12] until a kidney transplantation can be performed. Since ccRCC evolves from the proximal tubular cells targeted by orellanine, we hypothesized that orellanine toxicity extends to ccRCC cells. To test our hypothesis the effect of orellanine was determined in primary and established cell lines of ccRCC and in a new *in vivo* model of subcutaneous ccRCC based on nude rats on peritoneal dialysis.

## RESULTS

### Orellanine selectively decreases viability in proximal tubular cells

**Figure 1:**
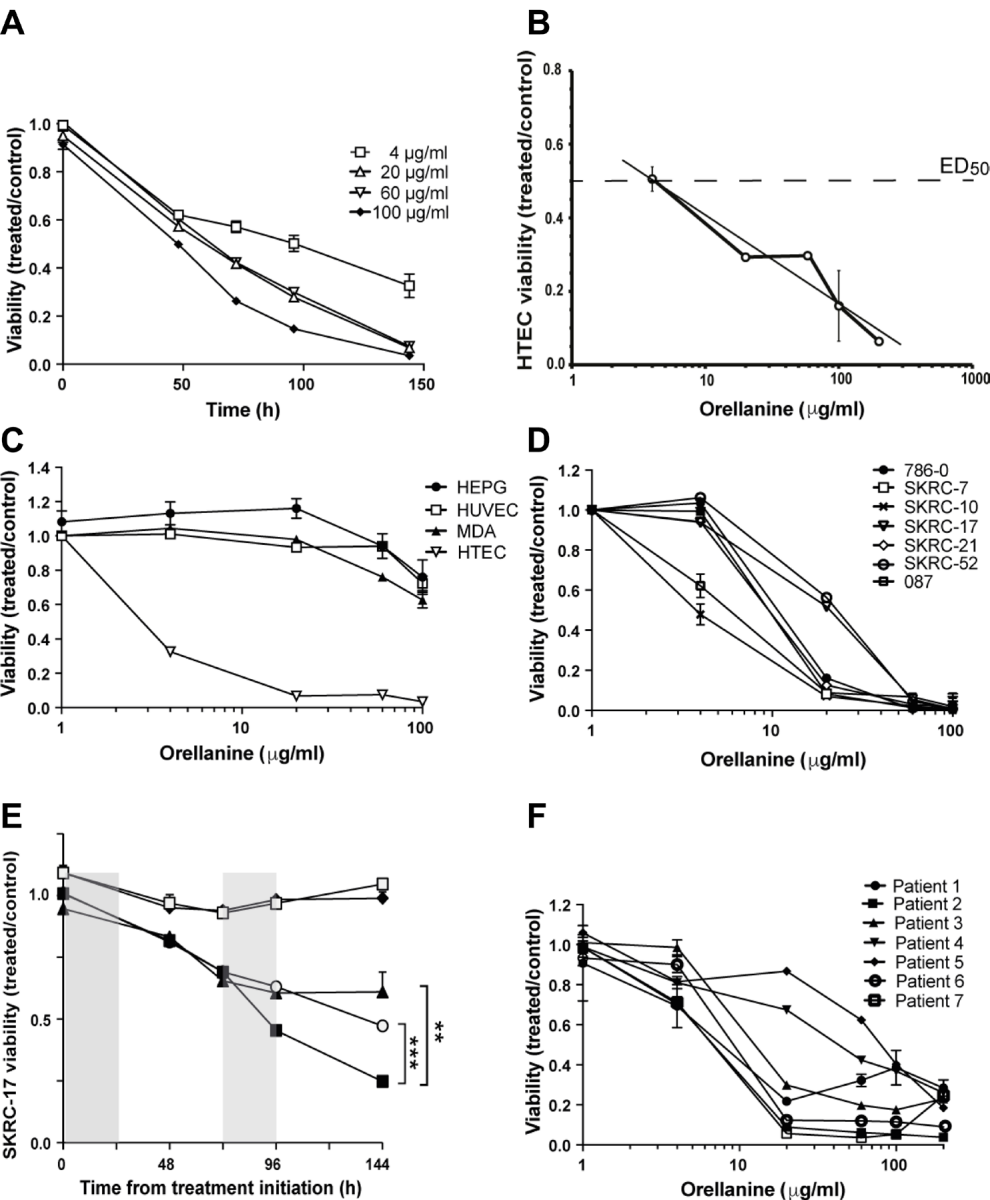
Orellanine is selectively toxic to human tubular epithelial cells and clear cell renal carcinoma cells (**A**) Viability of HTEC treated for 24 hours with orellanine, normalized to vehicle treated control (*n* = 6, mean ± SEM). (**B**) HTEC were exposed to different concentrations of orellanine for 24 hours and their viability was estimated using Alamar Blue technique at 72 hours, *n* = 6 for each data point. ED50 equals 4.1 ± 1.2 μg/ml. (**C**) Viability of HTEC, liver cells (HEPG2), breast cancer cells (MDA-MB-231) and HUVEC at 144 hours post 24 hour orellanine treatment (*n* = 6, mean ± SEM). (**D**) Viability of orellanine-treated ccRCC cell lines at 144 h, normalized to vehicle-treated controls (*n* = 6). One of the the two cell lines showing lowest sensitivity *in vitro* (SKRC-17 ) was chosen for the *in vivo* experiments. (**E**) The SKRC-17 cells were exposed to different concentrations of orellanine for 24 hours. The graphs represent repeated incubation at the doses (♦ 4 and ○ 20 μg/ml), single treatment (□ 4 and ▲ 20 μg/ml) and doubling of the incubation time from 24 to 48 hours (■ 20 μg/ml), respectively. (**F**) Orellanine treatment of primary renal cancer cells obtained from 7 patients with clear cell RCC. Data are presented as mean ± SEM and *p*-values are determined by one way ANOVA with Tukey’s post hoc test, where *p* < 0.05 was considered significant, **p < 0.01 ***, *p* < 0.001.

Human tubular epithelial cells (HTEC) are uniquely sensitive to orellanine [10]. Accordingly, exposure to orellanine caused irreversible damage to primary HTEC (Figure 1A) with an ED_50_ concentration of 4.1 ± 1.2 μg/ml (Figure 1B). In contrast, orellanine had only limited effects on human umbilical endothelial cells (HUVEC), hepatocytes (HEPG2), and a breast cancer cell line (MDA), as shown in Figure 1C. After removal of orellanine from the culture medium, HUVEC, MDA and HEPG recovered while the HTEC continued to die until no viable cells remained typically after several days. These results strongly support the notion that the toxicity of orellanine targets renal tubular epithelial cells in concentrations that leave other cells unaffected.

### The toxic effect of orellanine extends to human clear cell renal cancer cells

The unique susceptibility of proximal epithelial tubular cells to orellanine led us to hypothesize that they remain vulnerable even after transformation to cancer cells. In support of our hypothesis, orellanine induced a pronounced and dose-dependent decline in viability in renal cancer cell lines from primary tumors (786-O, SKRC-7, SKRC-10, SKRC-21, 087) and from metastatic lesions (SKRC-17, SKRC-52), as shown in Figure 1D. Repeated incubation at the lower doses did not yield any significant reduction in viability compared to single treatment. However, doubling the incubation time significantly reduced the viability (Figure 1E).

To determine the sensitivity of primary RCC cells to orellanine, fresh samples of renal tumors were obtained from 7 patients diagnosed with ccRCC. All primary renal cancer cells were susceptible to orellanine and responded similarly (Figure 1F) as the ccRCC cell lines (Figure 1D).

### Orellanine affects oxidative stress and cell metabolism

**Figure 2:**
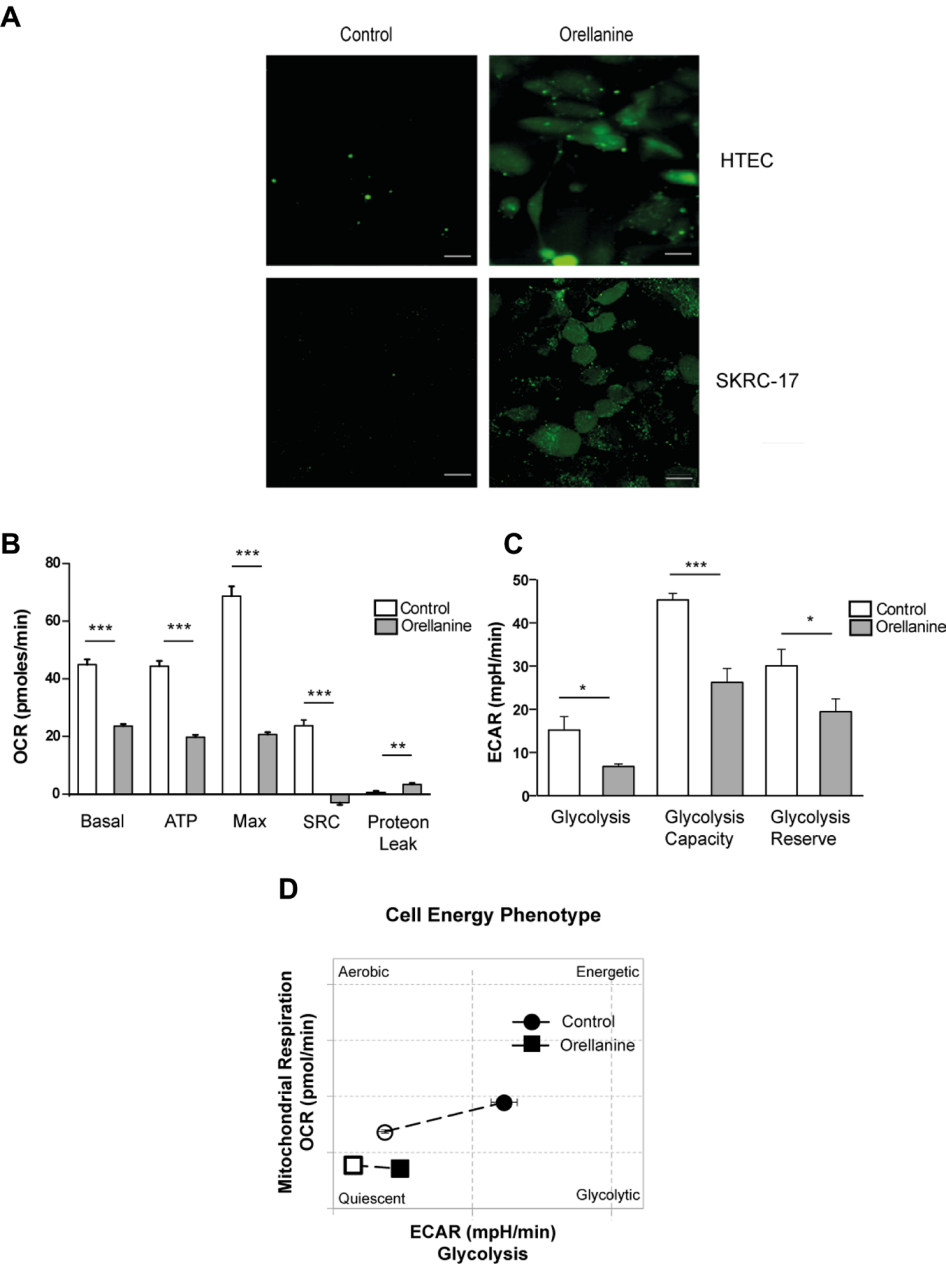
Orellanine induces oxidative stress and down-regulates cell metabolism (**A**) Oxidative stress in HTEC and SKRC-17 cells after 24 hours of vehicle or orellanine treatment, using carboxy-H2DCFDA for detection of ROS; scale bar 20 µm. Seahorse experiment showing: (**B**) Diagram of Basal Oxygen consumption rate (OCR) (basal), ATP production (ATP), maximum OCR (Max), spare respiratory capacity (SRC) and proton leak calculated from the OCR curve. (**C**) Glycolysis, glycolysis capacity and glycolysis reserve calculated from the Extracellular acidification rate (ECAR) curve. (**D**) Diagram showing the cell energy phenotype shift during mitochondrial stress conditions.

The toxic mechanism of orellanine is not fully understood but one of the proposed mechanisms of action is oxidative stress, since previous studies showed that orellanine promotes oxidative stress *in vivo* in renal tissue [11]. This lead us to explore whether this occurs also in isolated HTEC and in ccRCC cancer cells, *in vitro*. We could confirm that orellanine induces oxidative stress*,* visualized as an increase in ROS, both in HTEC and in the metastatic ccRCC cell line, SKRC-17 (Figure 2A).

To further uncover the toxic effect of orellanine we investigated its effect on cell metabolism. CcRCC are highly dependent on anaerobic glycolysis and thereby sensitive to inhibition of glycolysis [13], and we therefore analyzed mitochondrial respiration and glycolysis in orellanine-treated SKRC-17 cells. Using the Seahorse technique, we found that orellanine promoted mitochondrial dysfunction as revealed by decreased basal and maximum respiration capacity in combination with reduced ATP synthesis, spare respiratory capacity (SRC) and increased proton leakage (Figure 2B). Orellanine also reduced glycolytic utilization as indicated by decreased glycolysis, glycolytic capacity and glycolytic reserve (Figure 2C). Non-treated SKRC-17 cells shifted from mitochondrial respiration to glycolysis during stress conditions, while orellanine-treated cells showed low basal respiration and lacked the capacity to shift to glycolysis under stress conditions as shown in Figure 2D.

Taken together these data indicate that orellanine promotes both oxidative stress and interferes with the cell metabolism in metastasizing ccRCC.

### Orellanine promotes cell death

**Figure 3:**
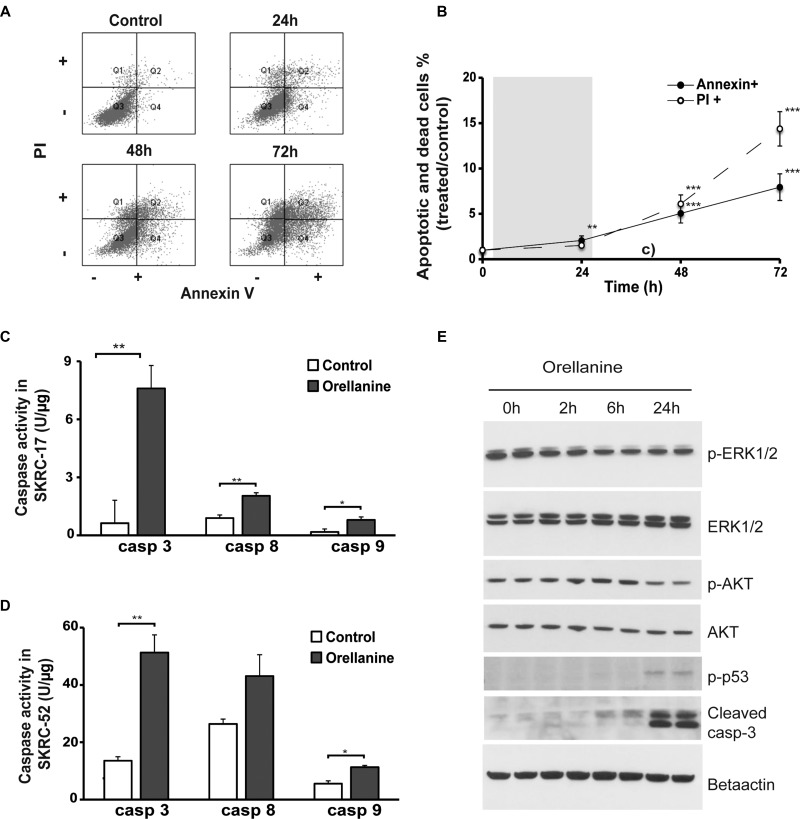
Orellanine promotes cell death in clear cell renal carcinoma cells (**A**) FACS scatter plots of vehicle or orellanine-treated SKRC-52 cells, treated for 24 hours with 100 μg orellanine/ml and analyzed 24, 48 and 72 hours post treatment initiation, for the presence of Annexin V and/or PI (*n* = 12, 9, 8 and 8 for controls, 24 h, 48 h and 72 h respectively). (**B**) The FACS plot presented graphically. PI indicates the cells in Q1 of Figure 3A (Necrosis). Annexin indicates PI+Annexin (late apoptosis) and Annexin (Early apoptosis), i.e. panel Q2 and Q4 in Figure 3A. Data are presented as mean +/- SEM and *p* values are determined by ANOVA with Tukey's post hoc test where *p* < 0.05 was considered significant. **p < 0.01, ***p < 0.001. Caspase 3, 8 and 9 activity in (**C**) SKRC-17 cells and (**D**) SKRC-52 cells treated with vehicle or 100 μg orellanine/ml for 24 hours, (*n* = 3, mean ± SEM, students *t*-test, *p < 0.05, **p < 0.01) (**E**) Western blots showing duplicate samples of total protein and phosphorylated p44/p42 MAPK (ERK1/2) (Thr202/Tyr204), AKT (Ser473), p53 (Ser15) and cleaved caspase-3 following 100 μg/ml orellanine exposure for 0, 2, 6 or 24 h. Beta actin served as a loading control.

To further understand how the toxic action of orellanine promotes programmed cell death in ccRCC we analyzed both survival and apoptotic signaling pathways. We examined the role of apoptosis and necrosis by performing Annexin/PI staining and FACS analysis on orellanine-treated SKRC-52 cells. A significant time-dependent increase in both apoptosis and necrosis was observed (Figure 3A–3B).

Caspase assays further confirmed up-regulation of apoptotic pathways in ccRCC following orellanine treatment, indicated by activation of both the extrinsic pathway (caspase 8) and the intrinsic pathway (caspase 9) in combination with increase of caspase 3, which is the caspase that both pathways converge into [14]. The effect was seen in both ccRCC cell lines tested, SKRC-17 (Figure 3C) and in SKRC-52 (Figure 3D). To further analyze induction of apoptosis and down-regulation of survival, we studied phosphorylation of proteins involved in survival and apoptotic pathways. ERK1 and 2 are known to be activated by phosphorylation and thereby regulate several processes such as cell cycle progression, cell adhesion, cell migration, transcription and survival. Following 2 h of incubation, orellanine reduced phosphorylated ERK1/2 activity. Another protein, AKT, is known to inactivate proteins in the apoptotic pathway (such as caspase 9) when phosphorylated at serine 473 [15]. Indeed, SKRC-17 cells treated with orellanine for 24 h showed decreased levels of phosphorylated AKT at s473, while total levels of AKT did not change. Since we know that orellanine is involved in ROS induction and DNA damage [11], we decided to examine phosphorylation of p53 at serine 15, which is the site of phosphorylation following DNA damage causing cell cycle arrest and apoptosis [16]. Orellanine induced a pronounced increase in phosphorylated p53 at s15 after 24 h of orellanine treatment. In addition, the apoptotic-inducer cleaved caspase-3 was increased after 24 h of orellanine treatment (Figure 3E).

To further evaluate the cytotoxic effect of orellanine on ccRCC cells, we used a proteomics approach. Several pathways were markedly altered in SKRC-17 cells treated with orellanine. The most strongly up-regulated pathway was *Cell Death* (apoptosis and necrosis) while the down-regulated pathways were protein synthesis, morphology, survival, movement, RNA posttranscriptional modification*,* development, growth and proliferation (see Table 1 for full list). All the above data support a strong toxicity of orellanine towards ccRCC.

**Table 1:** Cellular functions in SKRC-17 cells regulated by orellanine

Categories	Functions	Activation z-score	*p*-Value	Molecules
Cell Death and Survival	apoptosis	0.686	8.89E-07	NPM1,FN1,PPIA,ASNS,HNRNPK,EIF2A,DDX3X,CCT4,SRSF1,SOD2,FLNA,ANXA5,FASN,TCP1,ANXA7,APEX1,RAD23B,DHX9,RPS24,CALR,P4HB,TRIM28,PHB2,YWHAZ,CDK6,IGF2R,HNRNPA1,GAPDH,TFRC,SQSTM1,HSPB1
	cell death	0.534	3.75E-08	NPM1,FN1,PPIA,ASNS,HNRNPK,EIF2A,DDX3X,CCT4,EMG1,SRSF1,SOD2,FASN,TCP1,APEX1,RAD23B,DHX9,RPS24,CALR,P4HB,TRIM28,PHB2,YWHAZ,CDK6,LMNA,IGF2R,GPI,HNRNPA1,GAPDH,TFRC,SQSTM1,HSPB1
	necrosis	0.436	1.12E-07	NPM1,FN1,PPIA,ASNS,HNRNPK,EIF2A,DDX3X,CCT4,EMG1,SRSF1,SOD2,FLNA,FASN,TCP1,APEX1,MVP,RAD23B,RPS24,DHX9,CALR,P4HB,TRIM28,PHB2,YWHAZ,CDK6,LMNA,IGF2R,GPI,HNRNPA1,GAPDH,TFRC,SQSTM1,LDHA,HSPB1
Protein Synthesis	translation	−2.388	3.18E-13	CALR,EEF1A1,EIF3C,FN1,RPL23,MARS,EIF2A,HNRNPK,DDX3X,RPS7,PTBP1,GAPDH,APEX1,HSPB1
	synthesis	−3.042	4.06E-12	EEF1A1,NPM1,CALR,EIF3C,FN1,RPL23,MARS,EIF2A,HNRNPK,DDX3X,RPS7,PTBP1,GAPDH,APEX1,HSPB1
	metabolism	−2.854	1.48E-10	EEF1A1,NPM1,CALR,EIF3C,FN1,PFN1,ERAP1,PHB2,RPL23,MARS,CSTB,HNRNPK,EIF2A,DDX3X,RPS7,PTBP1,FLNA,GAPDH,SQSTM1,APEX1,HSPB1
Survival	survival	−1.947	1.51E-06	CALR,EIF3C,AGPS,PSMA6,P4HB,FN1,TRIM28,CDK6,PHB2,PPIA,PFDN2,IGF2R,DDX3X,SOD2,FLNA,FASN,ANXA5,EIF3A,TCP1,SQSTM1,APEX1,MVP
Cell Morphology	shape change	−1.982	7.81E-06	FN1,SOD2,FLNA,VIM
Cellular Movement	cell movement	−1.518	9.86E-05	NPM1,PFN1,FN1,HNRNPA2B1,CDK6,PHB2,YWHAZ,PPIA,VIM,TLN1,TPM1,IQGAP1,ASNS,HNRNPK,SRSF1,GPI,SOD2,FLNA,KARS,EIF3A,SQSTM1,HSPB1
RNA Post-Transcriptional Modification	processing	−1.387	4.90E-07	RPS7,SRSF1,PTBP1,NPM1,DDX5,HNRNPA1,HNRNPA2B1,HNRNPH3,HNRNPK,RPS24,HNRNPM
Cellular Development	proliferation	−0.583	1.74E-07	EEF1A1,NPM1,EIF3C,FN1,PFN1,MAP1B,HNRNPA2B1,TPM1,ASNS,IQGAP1,HNRNPK,DDX5,SOD2,FLNA,FASN,EIF3A,TCP1,ANXA7,APEX1,TRIM28,YWHAZ,CDK6,IGF2R,PTBP1,PRPS2,SSBP1,HNRNPA1,GAPDH,TFRC,LDHA
Cellular Growth and Proliferation	proliferation	−0.498	2.66E-10	CAPZA1,EEF1A1,NPM1,EIF3C,FN1,PFN1,MAP1B,HNRNPA2B1,PPIA,TPM1,IQGAP1,ASNS,HNRNPK,DDX3X,SRSF1,DDX5,SOD2,FLNA,FASN,KARS,EIF3A,TCP1,ANXA7,APEX1,MVP,AHCY,CALR,TRIM28,CDK6,YWHAZ,MAGED2,LMNA,G3BP1,IGF2R,HNRNPM,PTBP1,PRPS2,GPI,HNRNPAB,SSBP1,HNRNPA1,SERPINH1,GAPDH,TFRC,PSAP,SQSTM1,LDHA

The significant (*p* < 10^−5^) numbers of regulated proteins are compared to the total number of existing proteins in each pathway (regulated/total). The activation z-score is calculated using the IPA analysis tool and predicts the activity pattern of the cellular function identified, positive numbers indicates increased activity and negative numbers decreased activity. The significant regulated molecules detected in each category is listen with their gene names.

### A new rodent chronic peritoneal dialysis model enables assessment of the antitumor effect of orellanine

**Figure 4:**
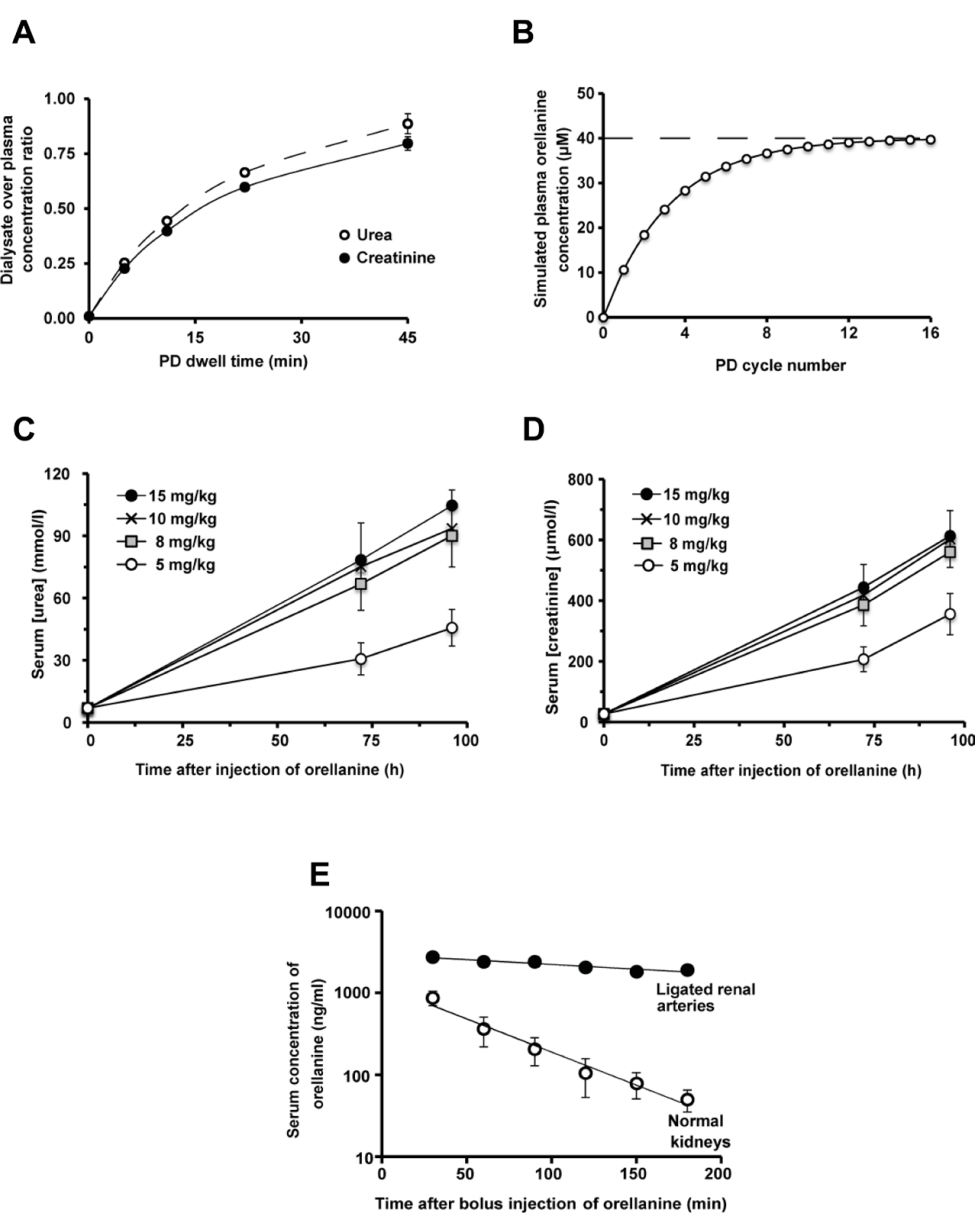
PD Dialysis and the effect of orellanine on kidney function in rat (**A**) The measured concentration of urea (full curve) and creatinine (dotted curve) in dialysate over plasma (D/P) concentrations in orellanine-treated anuric RNU-rats (*n* = 5) (**B**) Steady state serum orellanine concentration in rats weighing 150 grams, treated repeatedly with 15 ml dialysis solution containing 40 µM (i.e. 10 mg/L) of orellanine for 45 minutes at each cycle. The Figure is based on a kinetic modeling using an estimated D/D_0_ of 0.4 for orellanine after 45 minutes of dialysis. (**C**) Serum urea and (**D**) creatinine in subcutaneously orellanine treated Sprague Dawley rats, 72 hours post treatment at the doses indicated (*n* = 3 for each dose). (**E**) Orellanine concentration in serum over time after a bolus injection of 10 mg/kg orellanine intravenously in anesthetized RNU rats with ccRCC metastases during control and in rats with ligated renal arteries after a 10 mg/kg intravenous bolus dose of orellanine. Data are presented as mean as mean ± SEM and *p*-values are determined by *t* test, where *p* < 0.05 was considered significant. *p < 0.05.

To be able to study the effect of orellanine on ccRCC tumors we developed I) a chronic rat peritoneal dialysis (PD) model and II) a human ccRCC xenograft athymic nude rat model. These were combined in order to analyze the anti-tumor effect of orellanine. The efficiency of the dialysis in the rat model was demonstrated by the dialysate to plasma concentration ratio (D/P). After a dwell time of 45 minutes the D/P for urea was 0.89 ± 0.05 while the D/P for creatinine was 0.80 ± 0.03. The efficiency of the PD was shown by dialysate urea and creatinine concentrations approaching those in plasma within 30 minutes (Figure 4A). In subsequent *in vivo* experiments, orellanine was added to the PD fluid in order to maintain a stable plasma concentration. As seen in Figure 4B, a steady-state serum concentration of orellanine was obtained within the first day. Orellanine administration resulted in renal failure as shown by a rapid rise in serum urea (Figure 4C) and creatinine (Figure 4D), with polyuria at day 2–3 that progressed into anuria at day 4–5 (male Sprague-Dawley rats). We also studied the elimination of orellanine after a bolus injection in anesthetized tumor-bearing RNU rats. The halflife was 37 ± 3 min in intact animals, and 259 ± 71 min in rats with ligated renal arteries indicating the kidney as main elimination route (Figure 4E).

### Orellanine markedly reduces viable tumor mass *in vivo* in rats carrying human renal cancer metastases

**Figure 5:**
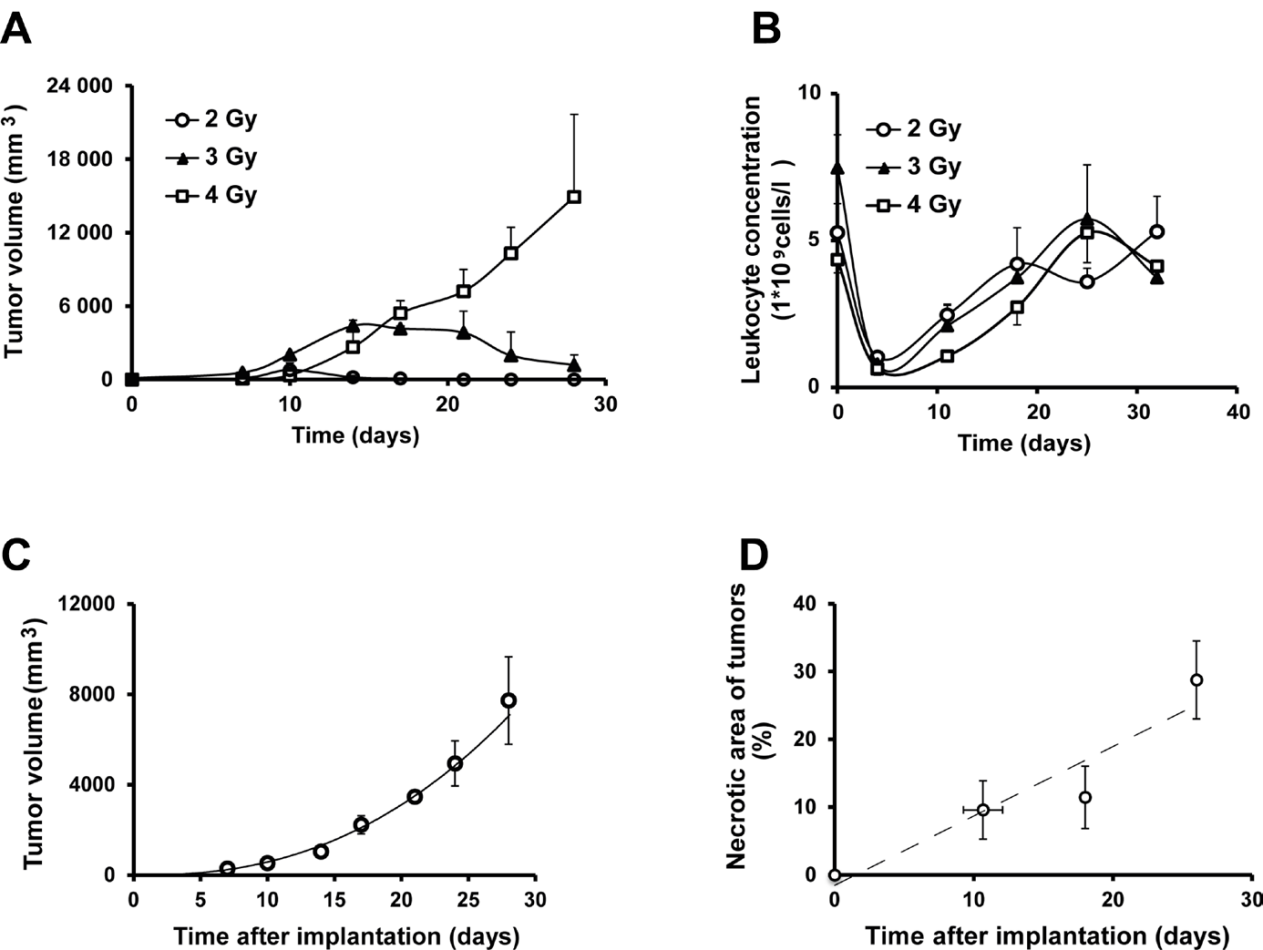
The human ccRCC xenograft model (**A**) Tumor growth of a xenograft model of human renal cancer (SKRC-17) in whole body irradiated RNU-rats at different radiation doses (*n* = 3 for each dose) (**B**) Leukocyte blood count post whole body irradiation of RNU-rats (*n* = 3). (**C**) Tumor volume in RNU-rats radiated with 5 gray 4 days pre inoculation of SKRC-17 cells subcutaneously (*n* = 3). (**D**) Necrotic area (%) over time in hematoxylin and eosin stained tumor sections from RNU-rats. (*n* = 6 at day 11, 7 at day 18 and 12 at day 26).

To establish a renal cancer model in rat we defined a radiation dose to suppress the B cell-mediated immune response against the xenografted tumor cells. A pre-implantation irradiation dose of 4 Gray provided a sustained growth of xenografted human renal cancer cells (SKRC-17, Figure 5A). Four days post-irradiation, when blood leukocyte count was lowest (Figure 5B), 10^7^ SKRC-17 tumor cells were implanted subcutaneously in the shoulder region. Tumor growth was measured weekly and the volume was calculated: (h*w*d*π/3), Figure 5C. The tumors showed 10% necrotic areas during the first two weeks, but as tumor volume increased, histologically determined necrosis in central parts of the tumors approached 30% (Figure 5D).

**Figure 6:**
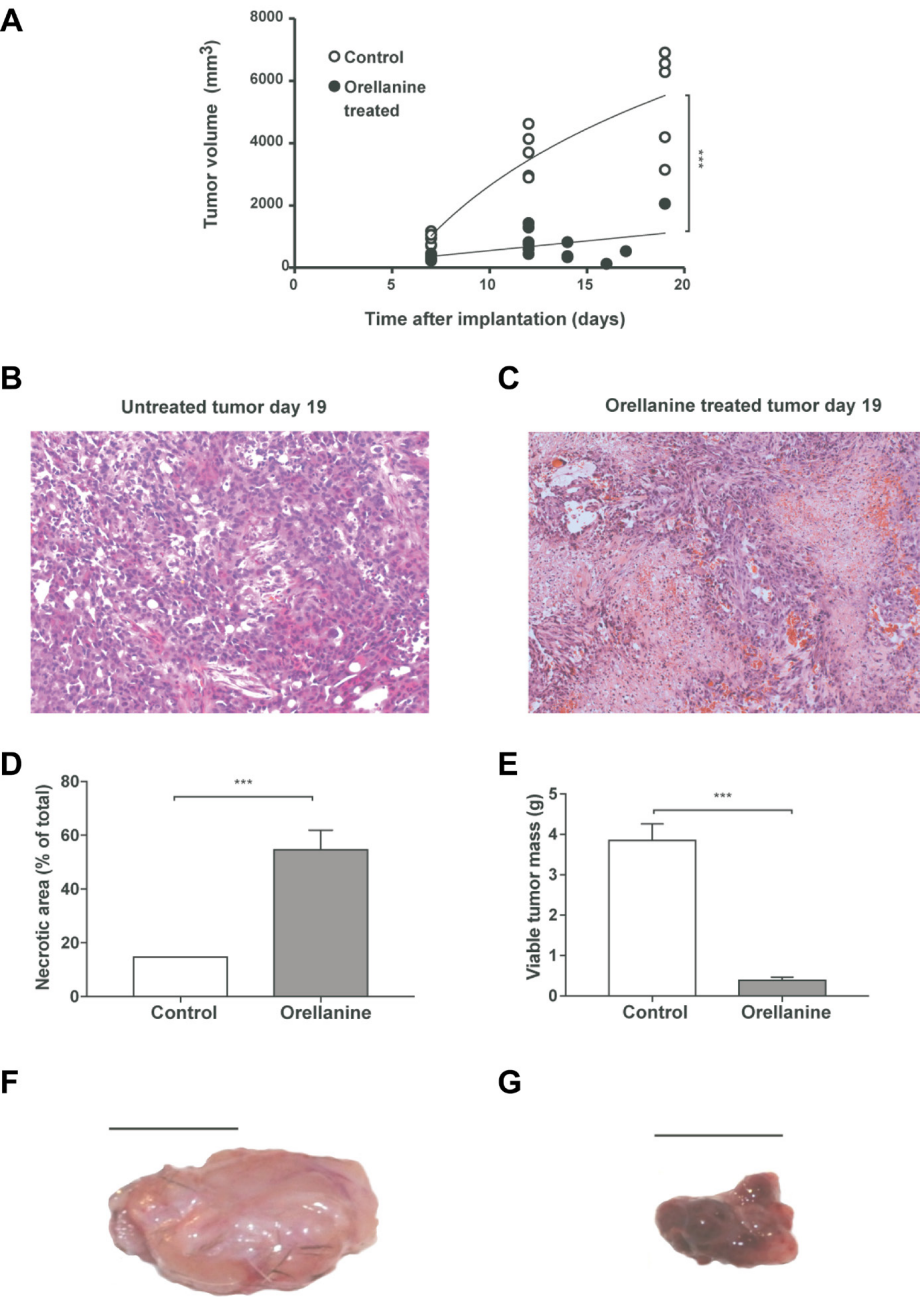
Orellanine significantly reduces tumor growth and induces necrosis Tumor volume in control rats and in rats receiving orellanine treatment, 10 mg/L for 48 hours via the dialysis solution at Day 8–9 (controls *n* = 5, treated *n* = 6). (**B**–**C**) Representative photos of hematoxylin and eosin stained tumor sections of control rats (B) and orellanine-treated rats (C), analyzed at day 16 post inoculation. (**D**) Necrotic area (%) of total tumor area and (**E**) viable tumor mass after subtraction of necrotic areas (controls *n* = 5, treated *n* = 6). (**F**) Representative photo of (F) control tumor and (**G**) orellanine-treated tumor. Data in a, d–e are presented as mean ± SEM and *p*-values are determined by *t* test, where *p* < 0.05 was considered significant. ***p < 0.001.

Tumor-bearing rats were treated with orellanine 10 mg/L intraperitoneally via the PD-solution. Treatment was initiated 7–8 days after subcutaneous implantation of SKRC-17 tumor cells. The tumors were growing stably for at least two weeks in control rats while orellanine treatment significantly inhibited tumor growth (Figure 6A). Eight days after initiation of orellanine treatment the necrotic areas covered roughly 15% in all control tumors (Figure 6B) but close to 55% in the orellanine-treated tumors (Figure 6C). Calculations of necrotic areas of the tumors showed almost 400% increase of necrosis in the orellanine-treated group compared to controls (Figure 6D), and more than 90% reduction of viable tumor mass (Figure 6E). The effect of treatment was particularly evident from the gross morphology of the tumors; they were large, pale and hard in the control group (Figure 6F), and small, purple and soft in the orellanine-treated group (Figure 6G).

**Figure 7:**
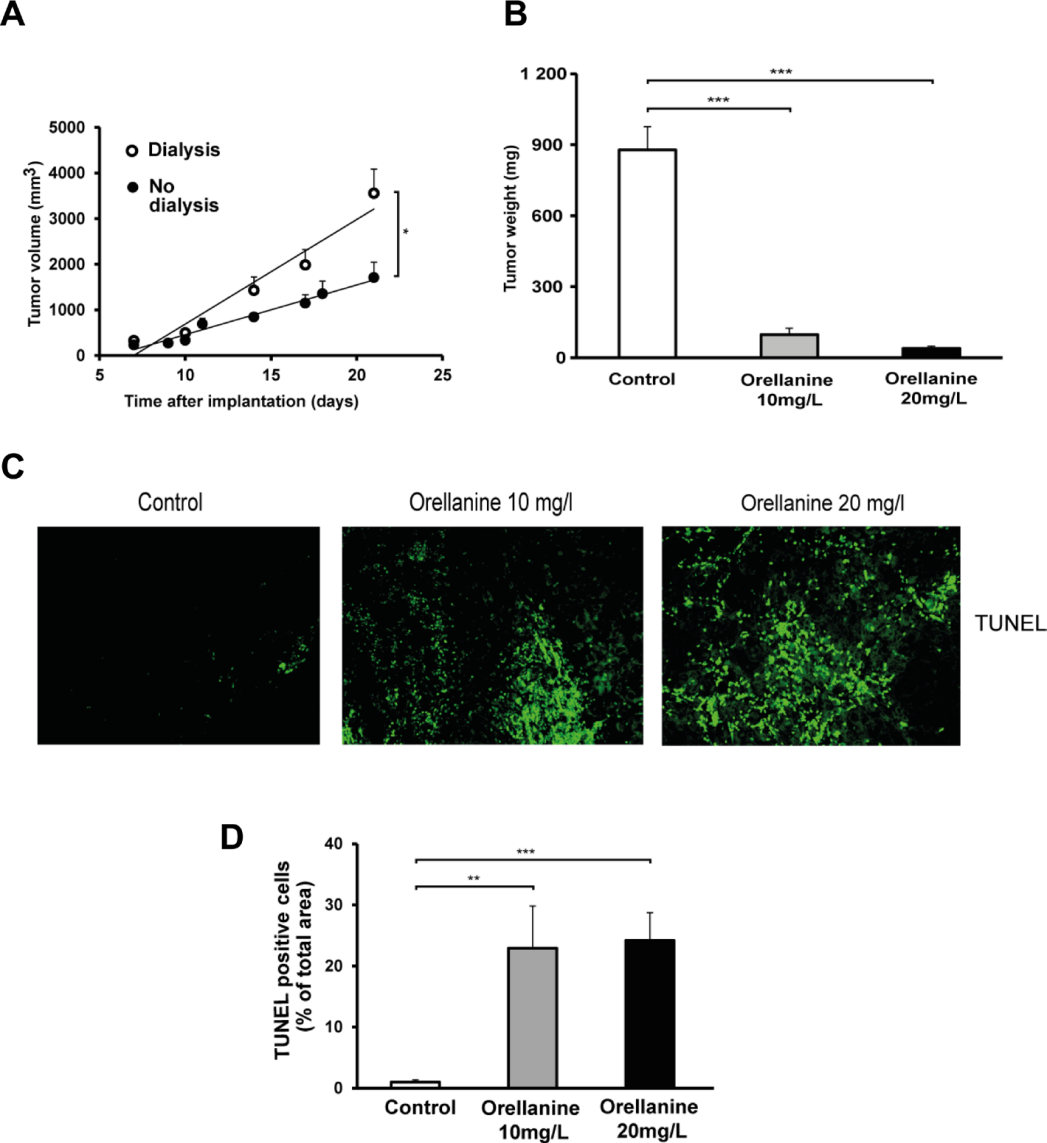
Orellanine-treated tumors are considerably smaller and display an evident increase in apoptosis (**A**) Tumor volume in RNU-rats, with or without one 15ml cycle of dialysis per day, irradiated with 4 gray 4 days pre implantation of SKRC-17 cells subcutaneously (*n* = 6). (**B**) Tumor weight of vechicle treated control tumors (*n* = 5) and tumors exposed to orellanine for 5 days (10 mg/L, *n* = 5) or 3 days (20 mg/L, *n* = 5). (**C**) TUNEL staining in tumor sections. From left; control, 10 mg/L orellanine-treated tumors and 20 mg/L orellanine-treated tumors. (**D**) Diagram showing TUNEL-positive cells in tumor sections (*n* = 25 for each concentration and *n* = 20 for control sections) from control tumors and tumors treated with 10 mg/L or 20 mg/L of orellanine. Data are presented as mean ± SEM and *p*-values are determined by one way ANOVA with Tukey’s post hoc test, where *p < 0.05 was considered significant. **p < 0.01, ***p < 0.001.

In another protocol we treated the tumors at an earlier stage (day 4 after implantation) with orellanine in the PD fluid, either with 10 mg/l for 5 days or 20 mg/l for 3 days. During this study we observed that dialysis *per se* increased tumor growth (Figure 7A). Despite the dialysis effect on tumor growth, markedly reduced tumor weight was seen already at day 4–6 after start of treatment (Figure 7B). In two rats the tumors were completely eliminated. In addition, TUNEL-staining revealed a pronounced increase of apoptosis in orellanine-treated tumors in both orellanine-treated groups when compared to control (Figure 7C). Percentages of TUNEL positive cells in the tumors were 23 ± 7% and 24 ± 5% in comparison to control for 10 and 20 mg/L of orellanine treatment (Figure 7D), indicating an efficient elimination of cancer cells by the use of orellanine already after a short treatment period.

## DISCUSSION

In this study, the mushroom-derived nephrotoxin orellanine dramatically reduced, and even completely eliminated human metastatic renal cancer xenografts growing in immune-suppressed rats. Orellanine efficiently reduced the viability of numerous renal cancer cell lines and primary renal cancers obtained from patients undergoing partial or total nephrectomy, with no signs of unspecific toxicity either *in vitro* or *in vivo*. Orellanine exerts its anti-tumor effect by disrupting vital cell functions such as energy metabolism, protein synthesis and cellular growth. The result is decreased survival and induction of apoptosis in ccRCC cells.

Susceptibility to the specific toxic effect of orellanine in HTEC was retained in all cancer cells investigated – established ccRCC (metastasized and non-metastasized) cell lines as well as primary ccRCC cells prepared from patients (Figure 1D, 1F). The toxicity of orellanine induced a pronounced decrease in viability of ccRCC and HTEC that irreversibly progressed several days after a 24-hour exposure, with no recovery observed. In contrast, the control cells tested (HUVEC, HEPG2 and MDA) were not affected except at the highest dose of 100 μg/ml (Figure 1B), with the effect beeing transient since cells recovered when orellanine was removed from the medium. This is well in line with patient studies where accidental ingestion of the mushrooms rendered the patients anuric and dialysis-dependent, most likely in a dose-dependent manner, with no detectable side-effects [17]. In a small retrospective study 28 patients who accidentially ingested the deadly webcaps were compared to matched dialysis or transplant controls. We saw that both groups had similar life expectancy and died of similar causes [18]. This report further strengthens the findings in this paper, that the toxicity of orellanine specifically targets proximal tubular cells and cancer cells of the same origin. The specific mechanism behind the accumulation of orellanine in these cells is not yet fully understood but is a topic of ongoing investigations in our laboratory.

To understand the toxic action of orellanine in ccRCC, we analyzed several important cellular functions that may be affected. We confirmed our previous findings [11] that orellanine induces a pronounced increase in ROS (Figure 2A) and in addition, the alterations of ROS also correlated to disturbances in mitochondrial function (Figure 2B–2D). Accumulating evidence shows that mitochondrial bioenergetics are needed for tumorigenesis and that targeting mitochondrial function may be efficient in cancer therapy [19]. One way to do that would be to inhibit glycogenesis, which is a highly efficient way to induce cell death in ccRCC cells [13]. Using the Seahorse technique, we showed that orellanine significantly disrupts mitochondrial function as revealed by lowered mitochondrial respiration and glycolysis following orellanine exposure (Figure 2B–2D). DNA damage was demonstrated by the effect of orellanine on increased phosphorylated p53 at serine 15 (Figure 3E). This site is phosphorylated following DNA damage, resulting in stabilization of p53 and inhibition of p53s interaction with its negative regulator MDM2 [20]. One of the hallmarks of ROS generation and mitochondrial dysfunction is induction of apoptosis. This was observed in orellanine-treated ccRCC cells as increased caspase activity (Figure 3C–3D) and increased annexin staining (Figure 3A–3B). Further support for the toxic effect of orellanine on ccRCC cells comes from proteomic analysis, revealing disturbances in several important cellular functions, such as protein synthesis and proliferation, in combination with decreased survival and increased apoptosis (Table 1). Thus, orellanine is highly toxic to ccRCC cells by promoting ROS, DNA damage and downstream cellular functions favoring cell death. We still do not know exactly how orellanine promotes this imbalance in the cell, whether solely a result of its effect on free radical production or also by disturbing other vital proteins and signaling pathways in the cell. Resolving this issue is another future aim.

To find out whether orellanine is as selective *in vivo* as *in vitro*, and potentially useful in a future clinical setting, we determined its efficiency in a newly developed human metastasis-derived ccRCC (SKRC-17) xenograft rat model. Since no animal model was available, a rat model of ccRCC was developed in B-cell depleted, athymic nude rats. In these rats, orellanine treatment significantly reduced tumor size (Figures 6 and 7) as compared to control. No overt effects on brain, lung, heart, liver, intestine, or skeletal muscle were seen when assessed by an independent pathologist. The kidneys, on the other hand and as expected, exhibited extensive tubular epithelial damage leading to complete renal failure within a few days. It is clear that the effect and selectivity of orellanine seen *in vitro* extends to the *in vivo* setting, i.e. the tumor burden is significantly reduced without affecting other tissues apart from the kidneys. It should be noted that we have not followed these animals over an extensive period of time since uremia, immunodeficiency, irradiation, tumors, orellanine-treatment and chronic peritoneal dialysis make them extremely susceptible to infections. Furthermore, rodents are particularly sensitive to uremia *per se*. In fact, this is the first report of anuric rats surviving on chronic dialysis with sufficient efficacy to maintain body homeostasis [21, 22]. When considering the translation to human disease and discussing orellanine as a treatment for human metastastatic clear cell carcinoma, one side effect is obvious – complete loss of renal function. Thus, any future patients on orellanine therapy would become dependent on renal replacement therapy, at least until they can receive a renal transplant. Therefore, patients already dependent on dialysis and diagnosed with metastatic ccRCC would be an ideal target population to test the clinical safety and efficacy of orellanine.

In conclusion, the *in vitro* and *in vivo* data presented in this study support and strengthen the hypothesis presented in the introduction: Orellanine toxicity extends to all human renal cancer cells studied so far *in vitro* as well as to ccRCC tumor bearing rats *in vivo*. Other cells and organs are not affected, which is in line with patient observations. Treatment with orellanine therefore remains a potential future option for surviving metastatic renal cancer, initially among those already dependent on renal dialysis.

## MATERIALS AND METHODS

### Orellanine synthesis and properties

Orellanine was synthesized by Ramidus AB, IDEON Lund, Sweden, as a 99% pure, freeze-dried powder without detectable contaminations of its metabolites, orellinine or orelline [23]. The substance was stored in a desiccator and protected from light. Before use the orellanine was diluted in 1 M HCl and the pH was carefully raised to 7.5 by adding NaOH to obtain a stock solution of 10 mg/ml that was stored as 1000 μl aliquots at −80°C [11]. Purity was checked using nuclear magnetic resonance spectroscopy and concentrations in biological fluids were determined using liquid chromatography–mass spectrometry [24]. The chemical stability of orellanine was confirmed using ^1^H NMR by RedGlead Discovery, Lund, Sweden. Orellanine is stable if protected from light.

### Cell lines and primary cell cultures

The ccRCC cell lines used were: 786-0 (obtained from ATCC), SKRC-7, -10, -17, -21 (obtained from and characterized by E. Oosterwijk, Radboud University Nijmegen Medical Center, Nijmegen, The Netherlands) and SKRC-52 (established at Memorial Sloan-Kettering Cancer center) [25]. Other cell lines used were human breast cancer cells, MDA-MB-231 and HUVEC cells (human umbilical cord vein endothelial cells, Lonza LTD).

To obtain pure primary cell cultures of human tubular epithelial cells (HTEC) and renal cell carcinoma cells, we collected renal tissue samples from nephrectomies performed due to malignancy. All specimens were collected after informed consent from the patients. Ethical permit was obtained from the regional ethical committee in Gothenburg before starting any studies. Cancer tissue or healthy cortical renal tissue was placed in complete culture media, minced and incubated with 300 U/ml Collagenase IV and 200U/ml deoxyribonuclease type II (Sigma-Aldrich) in DMEM (Gibco) at 37°C over night, followed by trypsin-EDTA treatment for 5 min at 37°C. The suspension was then passed through 70 µm and 20 µm mesh filters, and the obtained cells were cultured in culture media and treated with Stem Pro Accutase (LifeTechnologies) when replated.

The SKRC cell lines were maintained in RPMI culture medium, 786-0 cells in Dulbecco´s modified Eagle´s medium (DMEM), primary ccRCC cells in DMEM:F12 1:1, human breast cancer cells, MDA-MB-231 in DMEM. All media contained 10% fetal bovine serum and Antibiotic-Antimycotic solution (PAA Laboratories GmbH). HUVEC cells (Lonza LTD) were cultured in EGM-2 bullet kit (Lonza LTD) on attachment factor (Life Technologies) coated plates.

### mRNA analysis of oxidative defense components

To investigate the effect of orellanine on antioxidant genes in HTEC we used Real Time RT-PCR. Total RNA was isolated from HTEC using the RNeasy minikit (Qiagen), according to the manufacturer’s recommendations. To analyze different genes we used TaqMan Assay–on–Demand on ABI 7700 Sequence Detection System (ABI). The expression data were normalized to an endogenous control, glyceraldehyde-3-phosphate dehydrogenase (GAPDH). All probes were purchased from Life Technologies.

### ROS detection using carboxy-H_*2*_DCFDA

In HTEC we used the Image-iT LIVE Green Reactive Oxygen Species Detection kit (Molecular probes, Eugene, OR, USA) according to the manufacturer’s recommendations and analyzed using a fluorescence microscope. In SKRC-52 cells, ROS was detected by incubating cells with 25mM carboxy-H_2_DFFDA (Life Technologies) at 37°C for 30 min before analyzing with Flow Cytometry and FACSDiva software (version 6.1.1; BD Biosciences).

### Caspase activity assay

SKRC-17 and SKRC-52 were stimulated with 100 µg/ml orellanine for 2, 6 or 24 hours. Cells were washed twice with ice cold PBS before lysis. The bioactivity of caspase-3, caspase-8 and -9 was measured with a Fluorometric Assay Kit (Abcam). In brief, equal concentrations of protein lysates were incubated with the DEVD-AFC (caspase-3 substrate), IETD-AFC (caspase-8 substrate) or the caspase-9 substrate LEHD-AFC. The fluorescence of the cleaved substrates was determined at an excitation wavelength of 400 nm and an emission wavelength of 505 nm, using a fluorescence plate reader (Spectra Max Gemini XS, Molecular Devices).

### Cell viability assay

On all cell lines except the primary cells, cell viability was measured using the mitochondrial activity assay Alamar Blue (Life Technologies). A baseline measurement was done before exposure of the cells to orellanine in various concentrations for 24 hours. The change in fluorescence in the medium was monitored using a fluorescence plate reader (Spectra Max Gemini XS, Molecular Devices). Due to decreased numbers of mitochondria in the primary human renal cell carcinoma cells, the viability was instead measured using an ATP Assay (ViaLight Plus Kit, Lonza) at 48 h and 144 h after the beginning of the treatment by orellanine. The luminescence was monitored on a luminometer (Lmax, Molecular Devices , Sunnyvale, CA) and treated cells were compared with untreated cells.

### Proteomic analysis

SKRC-17 cells treated with 100 µg/ml orellanine for 24h or vechicle were harvested after 48 h in 50 mM triethylammonium bicarbonate buffer with 2% sodium lauryl sulfate. Protein levels were determined with Pierceä BCA Protein Assay (Thermo Scientific). Equal amounts (50 µg of each sample) were reduced, alkylated and digested with trypsin. The peptides were then tagged with reagent TMT 6plex^®^ and purified using Pierce Strong Cation Exchange Spin Column and PepClean C18 spin columns according to the manufacturer’s instructions (Thermo Scientific). LC-MS/MS analysis was performed in positive mode on an Orbitrap Fusion Tribrid mass spectrometer interfaced to an Easy-nLC 1000 (Thermo Fisher Scientific) using reversed-phase C18 separation.

To identify the differentially expressed proteins identified in the LC-MS/MS analysis we ran a *t*-test between treatment and control. The corresponding *p*-values were corrected for multiple testing using Benjamini-Hochberg’s method, which controls for the false discovery rate. We used Ingenuity Pathway Analysis software to find the enriched diseases and bio-functions for our data set. To ensure only significantly expressed proteins inside the limit of detection of MS data are considered, we used a 0.05 cut-off for the corrected *p*-values and a 1.15 cut-off for the fold changes.

### Measurement of oxygen consumption rate (OCR) and extracellular acidification rate (ECAR)

OCR and ECAR measurements were performed using the Seahorse XFp extracellular flux analyzer (Seahorse Biosciences, North Billerica, MA). Briefly, SKRC-17 cells were treated with 100 µg/ml Orellanine for 24 h, plated onto XFp cell culture plates (Seahorse Biosciences, North Billerica, MA) at a density of 10,000/well (Lonza, Belgium). One hour before assay, growth medium in the wells of an XFp cell plate was switched to XF base medium (200 µl) supplemented with 25 mM glucose, 1 mM pyruvate, and 2 mM glutamine for OCR or 2 mM glutamine for ECAR. OCR was measured by sequential injections of 5 µM oligomycin, 1 µM carbonyl cyanide 4-trifluoromethoxy-phenylhydrazone and rotenone plus antimycin A. ECAR was measured under glucose-starved conditions. Basal glycolysis rate was determined by injecting glucose (10 mM), Glycolytic capacity by injecting oligomycin (5 µM). Finally, 2-deoxyglucose (2-DG) was injected at 50 mM to measure the non-glycolytic acidification.

### Annexin and propidium iodide staining

To determine the rate of apoptotic and necrotic cells following orellanine treatment, SK were treated for 24 hours with 100 µg orellanine/ml and were subsequently cultured in orellanine-free medium. Cells were harvested 24, 48 and 72 hours post treatment initiation and incubated according to the Vybrant^®^ Apoptosis Assay Kit #2 (Life Technologies, Stockholm, Sweden) protocol and analyzed using FACS.

### Peritoneal dialysis of anuric rats

All animal experiments were approved by the Local Ethical Board for animal research in Gothenburg. For most experiments we used male athymic nude rats (RNU, Charles River, Germany, 150 g). For testing dose dependent urea and creatinine increases we used male Sprague-Dawley rats (Charles River, Germany). Since orellanine induces renal failure within 2–3 days, an efficient renal replacement therapy was needed. We developed an automated peritoneal dialysis (PD) technique allowing a highly efficient dialysis regimen in rats. Orellanine-treated rats were supplied with an indwelling, heparin-coated polyurethane peritoneal dialysis catheter (CBAS-50, Instech Laboratories Inc., PA, USA). 1.5 cm of the tip was planted in the abdominal cavity and the remaining part of the catheter was tunneled up to the neck and connected to a dual Luer Lock harness (SAI-infusion technologies, USA). At initiation of surgery, rats were administered 3 mg ciprofloxacin per kg body weight, 10 mg orellanine per kg body weight and buprenorphine (Temgesic^®^, 0.3 mg/kg). After recovery two days later, dialysis was initiated with PD solution (Gambrosol^®^ trio 10) containing 1.5% glucose and supplemented with 650 units of heparin/l. Five to seven exchanges of 15 ml each were performed daily in the automated PD-system. Dwell time was 60 minutes for each cycle.

### Orellanine dosage, administration and elimination

To evaluate the dose-effect of orellanine on kidney function, orellanine was subcutaneously injected at doses of 5 mg/kg or higher in male Sprague-Dawley rats. Aliquots of the dialysate concentrations of urea and creatinine were repeatedly collected during the study. At the last day of dialysis, when the animals were terminated, plasma samples were collected in order to estimate the U/P concentration ratios for urea and creatinine.

To analyze serum levels of orellanine in animals with or without kidney function, male RNU-rats with ligated arteries or sham operated were anesthetized and injected intravenously with 10 mg orellanine per kg body weight. Blood samples were collected 5, 30, 60, 90, 120, 150 and 180 minutes post-injection. Plasma concentration of orellanine was analyzed using a LC-MS/MS as described previously [24]. Rat plasma samples with known orellanine concentrations were used as a standard curve.

### Renal cancer tumor model

Four weeks old athymic male rats (RNU, Charles River, Germany) were radiated with a pre-implantation irradiation dose of 4 Gray to suppress also the B cell-mediated immune response. Four days post-irradiation 10^7^ SKRC-17 tumor cells were implanted subcutaneously in the shoulder region. Rats were randomized 1–3 or 7–8 days post-implant. At the earlier randomization point, where tumors where not yet measurable, rats were randomized according to body weight and in the latter, when tumors could be measured, according to tumor volume. Tumors were measured once or twice a week and the volume was calculated: V_t_ = (h*w*d*π/3).

### Histology

Tumors were measured, weighed and fixed in formalin and embedded in paraffin. Tumor sections of 5 µm were stained with hematoxylin and eosin. A pathologist determined the necrosis in % of the total tumor area, in a blinded manner.

### TUNEL staining

Staining for apoptotic cells was performed using a slight modification of the ApopTag method (Millipore). The terminal deoxynucleotide transferase (TdT) mixture (Millipore; ApopTag kit) was prepared according to the manufacturer’s instructions and incubated for 1 hour at 37°C. Anti-Digoxigenin-conjugate was then applied and the sections were incubated for 1 hour at room temperature. Sections were finally washed in PBS and mounted in ProLong Gold Antifade with DAPI nuclear staining (Life Technologies, Stockholm, Sweden).

### Statistics

Results are presented as mean ± standard error of the mean. Differences between groups were determined using parametric tests, Student’s *t*-test, ANOVA with Tukey’s or Dunnett’s Post Hoc Test or repeated measures ANOVA.
